# Life-course and Cohort Trajectories of Chronic Non-communicable Diseases Risk Factors in China

**Published:** 2017-05

**Authors:** Fan YANG, D. QIAN, Dan HU

**Affiliations:** School for Health Policy & Management, Nanjing Medical University, Hanzhong Road 140, Nanjing 210029, Jiangsu Province, P.R. CHINA

**Keywords:** Chronic non-communicable diseases, Risk factors, China, Life course

## Abstract

**Background:**

NCDs are the leading disease burdens in China and the NCDs risk factors shifts have accelerated at an unprecedented scale over the past 30 years. The aim of this study was to analysis the natural trajectories of NCDs risk factors over the life course.

**Methods:**

The large-scale longitudinal data from the CHNS includes nine rounds of surveys between 1989 and 2011. Overall, 145913 observations (29719 individuals) at multiple exams have been followed up over a 23-year period. The mixed-effects models with random intercepts were used to the characterize shifts in the distribution of these risk factors across the whole life course.

**Results:**

During about 23 years observational period across all age bands, the mean AMC, UAC, TSF, BMI, WC, DBP, SD, DD, and PA trajectory all increased until a certain age. Then decreased in both gender, whereas SBP strictly increased across lifespan; and the secular trend in AMC and WC, SBP, DBP was greater in women than in men; younger generations had higher AMC, UAC, TSF, BMI, WC, WHR, WHtR, SBP, DBP levels across adulthood, whereas younger birth cohorts had lower SD, DD, and PA levels.

**Conclusion:**

We observed in a large and comprehensive longitudinal dataset that provided strong evidence of population-wide secular shifts from childhood onwards, which suggests that promoting healthier lifestyles, body weight, blood pressure and enhancing the primary practitioner’s capability should be required to reduce the burden of NCDs in China.

## Introduction

Chronic non-communicable diseases (NCDs) are the leading disease burdens in China (1). Major causes leading to severe NCDs are the importance of changing risk factors that include reduced physical activities, higher intake of alcohol, increasing rates of smoking, obesity, and hypertension (2). These risk factors shifts have accelerated at an unprecedented scale in this developing country, accompanying rapid population ageing and continuing changes in socio-economic development over the past 30 yr (1).

Having no modifiable risk factors (3) and adherence to these was associated with lower risk of NCDs and lower mortality (4), therefore, keeping healthy behaviors, body weight and blood pressure are critical to healthy aging when the rapid transition of China’s population from a youthful to an ageing population. Moreover, NCDs often start in early life and their processes expand over the life course, the rate of these changes in risk factors and the early age at which they begin, are of especial concern. A better knowledge on the natural trajectories of these risk factors when life course approach ageing could provide essential information necessary for reducing the burden of age-associated diseases NCDs and then maintaining a healthy aging population (5). However, the cross-sectional surveys that based on data from repeated investigation and measurement are difficult to disentangle the effects of aging on NCDs risk factors. The large population-based cohorts may be used to determine whether patterns of NCDs risk factors across the life course represent true age and cohort effects. Nevertheless, to date, many studies only examined whether determinants of NCDs (6–8) or describe short-term trajectories of body mass index for adults aged 20–60 (9, 10); no previous study has jointly evaluated the natural trajectory of NCDs risk factors across whole lifespan.

Therefore, we used the China Health and Nutrition Survey (CHNS), a large general-purpose epidemiological population-based cohort and including participants followed up over a 23-yr period, to the characterize shifts in the distribution of these risk factors across the whole life course and examine modification of this effect by cohort and gender.

## Methods

This study used large-scale longitudinal data from the CHNS that was a prospective household-based study, including multiple ages and cohorts across nine diverse provinces and nine rounds of surveys between 1989 and 2011.

Survey protocols, instruments and the process for obtaining informed consent for this study were approved by the Institutional Review Committees of the University of North Carolina at Chapel Hill, the National Institute of Nutrition and Food Safety, Chinese Center for Disease Control and Prevention, and the China-Japan Friendship Hospital, Ministry of Health.

The CHNS was designed to cover key public health risk factors and health outcomes, demographic, social and economic factors in depth at the individual, household and community levels, covering approximately 56% of China’s population, including Heilongjiang, Liaoning, Guangxi, Guizhou, Hubei, Hunan, Henan, Jiangsu, and Shandong. The CHNS waves were conducted in 1989, 1991, 1993, 1997, 2000, 2004, 2006, 2009 and 2011. A multistage, random cluster process was used to draw the sample in each of the provinces. Counties and cities in each province were stratified by income (low, middle and high) and a weighted sampling scheme was used to select randomly four counties and two cities in each province. Villages and townships within the counties and urban and suburban neighborhoods within the cities were selected randomly. In each community, 20 households were randomly selected and all household members were interviewed; Additional details of sampling, response rates, and data quality are reported elsewhere (11, 12). Participants were eligible for analysis if they were 7 yr or older, resulting in an analytic sample of 145913 observations (29719 individuals) at multiple exams (mean number of exams: 5).

Data was collected at the participants’ houses or a local clinic by well-trained health workers and followed the same protocol. Height and weight were measured without shoes to the nearest 0.1 cm or o.1 kg by trained health workers who followed standardized procedures using calibrated equipment (SECA 206 wall-mounted metal tapes and SECA 880 scales). Body Mass Index (BMI, kg/m^2^) was calculated as the ratio of weight (in kg) and height-squared (in m^2^). The method (13) was used to measure triceps skinfold thickness (TSF). The average values of the three measurements were used in our data analysis. The upper arm circumference (UAC) was measured at the midpoint of the upper left arm between the acromion process and the tip of the olecranon. Arm muscle circumference (AMC) was calculated using the standard formula (14): AMC = UAC-(3.14*TSF). Waist circumference (WC) was measured with a Seca tape measure (Seca North America, Chino, CA, USA) at the midpoint between the lowest rib margin and the iliac crest on the midaxillary and the inter-iliac crest lines and hip circumference was measured at the level of maximal gluteal protrusion. Waist-hip ratio (WHR) and waist-height ratio (WHtR) were determined from waist circumference (cm) divided by hip circumference (cm) and height (cm), respectively (15). Seated systolic/diastolic BP was measured three times on the right arm by trained technicians in triplicate after a 10-min rest, using mercury sphygmomanometers with appropriate cuff sizes (16). The average values of the three measurements were used in our data analysis. The indicator for smoking does (SD) was based on responses to the survey item, “Do you still smoke cigarettes (including hand-rolled or device-rolled) now: yes or no”. “How many cigarettes do you smoke per day?” Drinking does (DD) was based on responses to the survey item, “Do you drink this type of alcohol (Beer, Grape wine including various colored wines, rice wine, Liquor) ?” “How much do you drink each week?” The total amount consumed was calculated as grams (g) of pure alcohol, based on the beverage type and amount drunk. Physical activity (Total metabolic equivalents, hours/week): a detailed weekly physical activity recall spanning one year was assessed using questionnaires that probed the time spent in a typical week at work, in travel, in leisure time and in domestic activity. Total MET minutes at work, in travel, in leisure time and in domestic activity were summed using minutes spent in each activity multiplied by metabolic equivalents (METs) for that activity (17).

Quadratic age-related NCDs risk factors trajectories for the mean were assessed by mixed-effects models with random intercepts. All models were calculated separately for men and women to assess explicitly age and cohort effect in NCDs risk factors and estimated using a maximum likelihood algorithm. Changes in NCDs Risk Factors by age were calculated from observed data within each cohort and represented in terms of the mean percentage of individuals reporting a particular category of consumption at any given age. The model used can be formulated as follows:

$$Y_{\mathrm{ti}}=\beta_{0i}+\beta_{1i}{\mathrm{Age}}_{\mathrm{ti}}+\beta_{2i}{{\mathrm{Age}}_{\mathrm{ti}}}^{2}+\beta_{3i}{\mathrm{cohort}}_{\mathrm{ti}}*{\mathrm{Age}}_{\mathrm{ti}}+\beta_{4i}{\mathrm{cohort}}_{\mathrm{ti}}*{{\mathrm{Age}}_{\mathrm{ti}}}^{2}+e_{\mathrm{ti}}$$

where Y_ti_ is the measurement instance for any given risk factor for subject i at time t; *β*_0,_ *β*_1_ . . . , *β*_k_ are the population mean intercept and slopes for the explanatory variables and *e_ti_* is the random error within individuals over time. The coefficients in the Equation can be interpreted as the age and cohort effect. Descriptive characteristics are presented as means (standard deviations) and percentages. All statistical tests were 2-sided and were performed using STATA ver. 12.

## Results

The sample characteristics are shown in Table 1.

**Table 1:** Descriptive characteristics of population (age>=7) who participated in the 1989–2011 China Health and Nutrition Survey ^a^

	**Survey Year**								
	**1989**	**1991**	**1993**	**1997**	**2000**	**2004**	**2006**	**2009**	**2011**
Participated	13835	13737	13307	10971	12811	13453	13851	14975	15690
New participated		412	374	3849	3451	1971	4092	2987	6147
With drowal/Died		98	842	2710	2009	2809	1573	2968	2272
Gender	49.7	49.4	49.4	49.8	49.6	49.6	48.1	47.5	47.6
Age	33.4(18.1)	34.1(18.4)	34.5(18.6)	35.8(18.9)	37.0(18.8)	39.6(19.0)	39.8(18.3)	41.3(18.3)	42.5(18.5)
SBP	110.9(13.1)	110.6(19.1)	111.3(18.6)	114.3(19.3)	115.9(18.9)	119.7(19.4)	119.0(19.0)	122.2(19.7)	122.1(18.8)
DBP	72.5(9.4)	71.7(12.1)	72.8(11.9)	74.3(12.1)	75.3(11.9)	77.2(11.8)	77.3(11.6)	79.1(11.8)	77.9(11.3)
BMI	21.5(2.4)	20.7(3.4)	20.8(3.4)	21.2(3.7)	21.8(3.8)	22.4(3.8)	22.6(3.7)	22.8(3.8)	23.2(4.1)
AMC	21.1(2.9)	20.2(3.6)	20.7(3.5)	20.3(3.9)	20.4(3.9)	21.1(4.1)	21.0(4.0)	21.5(3.8)	21.7(4.1)
TSF	12.6(7.5)	10.7(6.2)	10.7(6.0)	11.8(6.7)	14.2(7.8)	14.6(7.8)	16.2(7.8)	15.9(7.5)	17.1(7.7)
UAC	25.0(2.5)	23.6(3.9)	23.5(4.0)	24.1(4.2)	24.8(4.3)	25.7(5.0)	26.1(4.8)	26.6(4.6)	27.1(4.8)
WC			73.8(10.2)	74.0(11.5)	76.2(11.8)	78.6(11.6)	79.2(11.7)	80.8(11.9)	82.0(12.3)
WHR			0.8(0.1)	0.8(0.1)	0.8(0.1)	0.9(0.1)	0.9(0.1)	0.9(0.1)	0.9(0.1)
WHtR			0.5(0.1)	0.5(0.1)	0.5(0.1)	0.5(0.1)	0.5(0.1)	0.5(0.1)	0.5(0.1)
SD^※^				5.0(8.8)	4.7(8.7)	4.6(8.6)	4.4(8.6)	4.6(9.0)	4.3(8.6)
DD^※^				8.6(22.9)	10.9(26.8)	9.2(25.1)	8.8(25.3)	7.5(21.0)	6.8(18.7)
PA^※^						159.4(138.6)	156.3(138.0)	151.4(132.3)	144.4(129.4)

a Values presented as numbers for arbitrary values and as mean±SD or % for other variables.

※ only collected form adult population (age>=18). //SBP, Systolic blood pressure; DBP, Diastolic blood pressure; BMI, Body mass index; mean arm muscle circumference, AMC; upper arm circumference, UAC; triceps skin fold thickness, TSF; Waist circumference, WC; Waist-hip ratio, WHR; waist-height ratio, WHtR; Smoking does, SD; Drinking does, DD; Physical activity, PA.

**Fig. 1:**
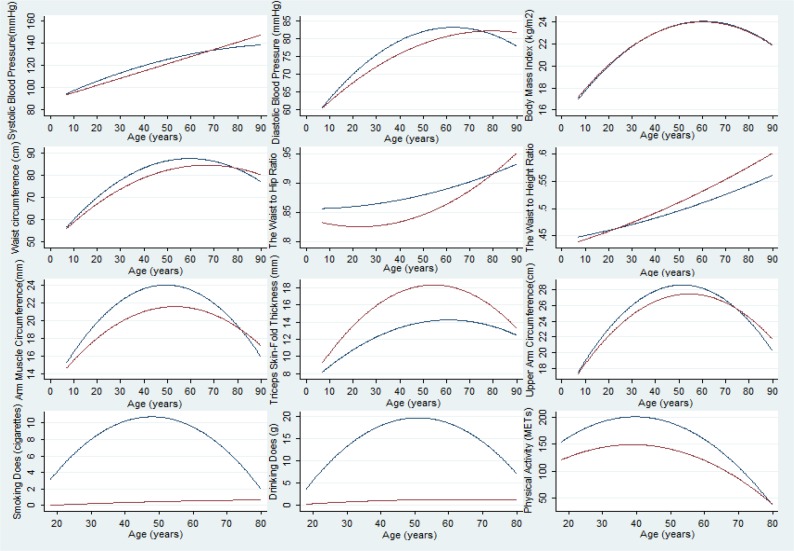
Predicted mean Chronic Non-communicable Diseases Risk Factors trajectories across the life course among men (blue) and women (red)

**Fig. 2:**
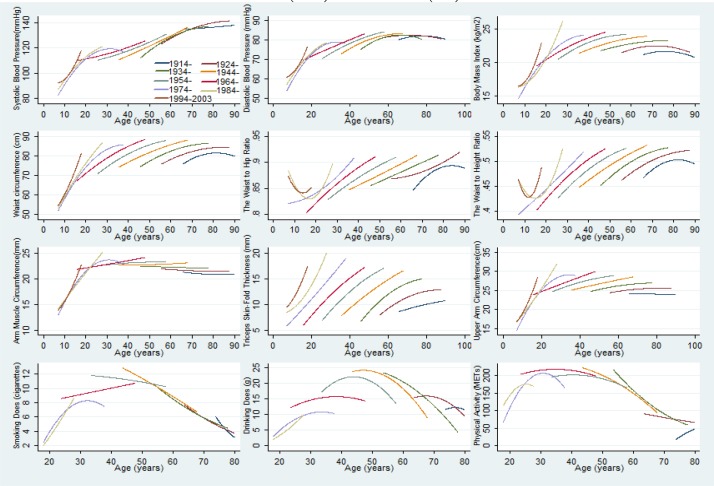
Predicted mean Chronic Non-communicable Diseases Risk Factors trajectories with adjustment for nine different birth cohorts among men

**Fig. 3:**
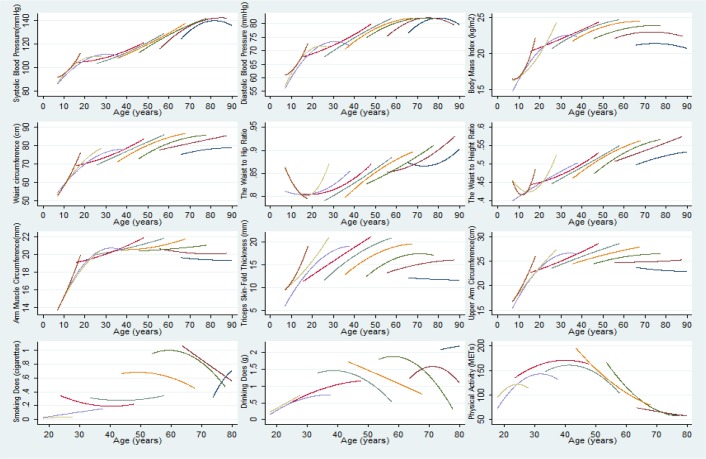
Predicted mean Chronic Non-communicable Diseases Risk Factors trajectories with adjustment for nine different birth cohorts among women

Age-related mean risk factor trajectories for the entire study population are displayed in Fig. 1, 2 and 3. The final model is presented separately for men (Table 2A) and for women (Table 2B). The mean SD and DD trajectory increased until age 51 and then decreased markedly in men, whereas SD and DD slightly increase with age in women (Fig. 1).

**Table 2:** **A, B:** Estimated coefficients in age-related trajectories (mixed-effects models) of Chronic Non-communicable Diseases Risk Factors for men (A) and women (B) (age>=18), respectively

**A**	**Intercept**	**Age**	**Age**^**2**^	**Cohort**	**Cohort×Age**	**Cohort×Age**^**2**^
SBP(mmHg)	66.28***	1.20***	−0.0042***	6.09***	−0.15***	0.0014***
DBP(mmHg)	31.94***	1.30***	−0.0087***	4.13***	−0.083***	0.00054***
BMI(kg/m^2^)	12.30***	0.21***	−0.0013***	0.45***	0.017***	−0.00018***
WC(cm)	34.05***	0.81***	−0.0031***	2.81***	0.057***	−0.00075***
WHR	0.7257***	0.0011	9.77E^−6^	0.0012	0.00066***	−6.53E^−6^***
WHtR	0.2509***	0.0038***	−1.00E^−5^*	0.0108***	0.00039***	−4.41E^−6^***
AMC(mm)	23.52***	−0.0888**	0.00063**	−0.6711***	0.04040***	−0.00032***
UAC(cm)	15.23***	0.1321***	−0.00047	0.5788***	0.03409***	−0.00029***
TSF(mm)	−24.47***	0.65***	−0.0031***	3.68***	−0.0120	−2.69E^−5^
SD(cigarettes)	19.68***	−0.0903	−0.0012	−3.93***	0.1282***	−0.0011***
DD (g)	−37.80***	1.58***	−0.0127***	1.63	0.0307	−0.00052
PA (METs)	416.17***	−6.04*	0.0254	−61.49***	2.52***	−0.0248***

**B**	**Intercept**	**Age**	**Age**^**2**^	**Cohort**	**Cohort×Age**	**Cohort×Age**^**2**^
SBP(mmHg)	61.02***	1.41***	−0.0064***	6.83***	−0.25***	0.0028***
DBP(mmHg)	37.37***	1.13***	−0.0077***	3.52***	−0.11***	0.0011***
BMI(kg/m^2^)	15.17***	0.22***	−0.0019***	0.38***	−0.009**	0.00025***
WC(cm)	41.21***	0.87***	−0.0057***	2.96***	−0.065***	0.0010***
WHR	0.65***	0.0036***	1.29E^−5^*	0.01749***	−0.00035***	4.95E^−6^***
WHtR	0.3044***	0.0046***	−2.65E^−5^***	0.0146***	−0.0004***	6.79E−^6^***
AMC(mm)	20.40***	−0.0268	0.000025	−0.5051***	−0.02147***	−5.32E^−5^
UAC(cm)	16.53***	0.1719***	−0.00144***	0.3498**	0.0116*	9.97E^−5^***
TSF(mm)	−9.74***	0.5400***	−0.0028***	2.3305***	−0.01612	0.00032***
SD (cigarettes)	−2.70***	0.1490***	−0.0013***	0.3954***	−0.0208***	0.00017***
DD (g)	−2.76	0.0813	−0.00028	0.0852	0.0090	−0.00017*
PA (METs)	469.84***	−9.86***	0.0630***	−69.58***	2.7049***	−0.0255***

* *P*<0.05,

** *P*<0.01,

*** *P*<0.001

SBP, Systolic blood pressure; DBP, Diastolic blood pressure; BMI, Body mass index; mean arm muscle circumference, AMC; upper arm circumference, UAC; triceps skin fold thickness, TSF; Waist circumference, WC; Waist-hip ratio, WHR; waist-height ratio, WHtR; Smoking does, SD; Drinking does, DD; Physical activity, PA.

The peaks of the adjusted SD and DD trajectories occurred at earlier ages in younger men generations, who also had generally lower SD and DD levels (Fig 2. and 3). Physical activity (Total metabolic equivalents, hours/week) increased until age 40, and then decreased markedly in both men and women (Fig. 1). The secular trend in PA was greater in men than in women and younger generations, except the 1945 cohorts and men in late adulthood, had lower PA levels (Fig. 2 and 3).

The mean AMC and UAC trajectory increased until age 50 then decreased markedly in both gender, and the secular trend in AMC and UAC was greater in men than in women (Fig. 1). Younger generations had lower AMC and UAC levels across adulthood (Fig. 2 and 3). The TSF trajectory in men increased up to age 55 before declining whereas for women the corresponding peak age was age 60; women had higher TSF levels during the entire age range (Fig. 1). Furthermore, at any given age (between ages 7 and 90), individuals who were younger birth cohorts had higher mean TSF compared with older generations (Fig. 2 and 3). Moreover, the mean BMI and WC trajectory increased until age 60 and then decreased in both men and women (Fig. 1). The peaks of the BMI and WC trajectories occurred at later ages in younger generations, who also had generally higher BMI and WC levels (Fig. 2 and 3). Women had a steeper increase trajectory in WHR and WHtR compared with men between ages 7 and 90: (0.12. vs 0.08), (0.16 vs 0.11), respectively (Fig. 1). Younger generations had higher WHR and WHtR levels and those born in later cohort had slightly declined between ages 7 and 12 (Fig. 2 and 3).

SBP increased faster with age in women than in men: 53mmHg vs 43 mmHg, respectively, between ages 7 and 90 (Fig. 1). Younger birth cohorts had higher mean SBP compared with older generations (Fig. 2 and 3). The DBP trajectory in men increased up to age 60, peaking at 83mmHg before declining whereas for women the corresponding peak value was 82 mmHg at age 80 (Fig. 1). The peaks of the adjusted DBP trajectories occurred at earlier ages in younger generations, but who also had generally higher DBP levels (Fig. 2 and 3).

## Discussion

In this study, we demonstrate a large and comprehensive longitudinal dataset that included participants whose have been followed up over a 23-yr period, in which we jointly evaluated the natural trajectories of these risk factors of NCDs across whole lifespan. Few datasets have the power to demonstrate definitively disentangle the effects of aging and cohort on these risk factors; the present study provides the first data determining whether patterns of NCDs risk factors gain over the life course represent true age and cohort effects. During about 23-yr observational period across all age bands, the mean AMC, UAC, TSF, BMI, WC, DBP, SD, DD, and PA trajectory all increased until a certain age then decreased in both gender whereas SBP strictly increased across lifespan, and the secular trend in AMC, TSF, WC, SBP, and DBP was greater in women than in men; younger generations had higher AMC, UAC, TSF, BMI, WC, WHR, WHtR, SBP and DBP levels across adulthood whereas younger birth cohorts had lower SD, DD, and PA levels.

### Unhealthy behaviors

In our longitudinal analyses, the mean SD trajectory increased until age 51 and then decreased markedly in men. In western countries, most studies assessed the trends of smoking behaviors from adolescence to adulthood, which showed smoking prevalence increased strongly between the ages of 15 and 21 yr, and stabilized after that; or cigarette smoking started to decline after 25 yr old (18, 19). Although our results cannot directly comparable with those surveys because our study only included smoking does. Society, economics, and culture effects on smoking in both men and women, and physiological, psychological, sociodemographic, personal, and sociocultural factors play specific roles in smoking behavior (20). The China National Hypertension Survey of 169871 Chinese adults who were 40 yr of age or older conducted in 1999 and 2000, proved that there were dose–response association between pack-years smoked and death from any cause in both men and women (21); tobacco use continues in our study to be one of the most serious public health concerns of our time.

DD increased until middle age among men and slightly increased across adulthood among women, which is not consistent with the result of other countries. In the United Kingdom, among men, mean drinking consumption rose sharply during adolescence, peaked at around age 25 yr at 20 units per week. Then declined and plateaued during midlife, similar mean trajectories were seen for women, but with lower overall consumption; In USA, people start drinking in adolescence, increased their drinking in young adulthood and decreased, especially heavy drinking, after the age of 30 (22). A nationally representative prospective cohort study has shown that drinking using have been linked to a strong heart disease, cancer, stroke, and respiratory disease (23). The crumb of comfort is that younger birth cohorts had lower SD and DD levels compared with older generations, which may suggest that public health education and preventive measures may play a certain effect in the prevention.

PA increased until age 40 and then decreased markedly in both men and women; which findings are consistent with other surveys. In other countries, many older adults are also physically inactive and such inactivity increases with age (24, 25). Physical activity could reduce the risk of coronary heart disease, stroke, hypertension, diabetes, and improve bone health and cognitive and physical function, particularly for older people (26). However, after entering aging, physical activity of adults in our study did not meet the recommendation that suggested elderly to perform at least 150 min of moderate intensity aerobic physical activity per week (26). Even more serious is that younger generations were more likely to have lower PA levels. This may be mainly due to the reduction of the agricultural sector, increase of light work-related physical activity and changes in acquisition of motor vehicles that brought by the modernization, which led to decline of physical activity at transport or work among Chinese adults. Therefore, our result is a reminder of the urgent need for continued strengthening of national programs in China on lower physical activity cessation, especially for younger generations and elderly.

### Obesity

In our study, the mean BMI and WC trajectory increased until age 60 and then decreased in both men and women. Younger generations have experienced higher BMI and WC at earlier ages than ever before, which findings are consistent with other countries. The BMI distribution became more right-skewed in Canadian, U.S. (27), and United Kingdom adult populations (28). The 1985–2009 Whitehall II longitudinal study documented BMI and WC both increased faster in younger birth cohorts and was at higher levels than in older generations. In addition, other china cross-sectional surveys also have shown BMI and WC rose across period (29), patterns of China pediatrics and adult obesity start to have similar patterns in these western countries (30). China modernization and economic development brought improvement in nutritional status, with reduction in underweight and an increase in obesity for Chinese children and adult and so the peaks of the BMI and WC trajectories occurred at later ages in Chinese younger generations, who also had generally higher BMI and WC levels. Higher BMI is a stronger predictor of metabolic syndrome, diabetes and coronary heart disease in China and increasing WC is an independent indicator of cardiometabolic risk, which calls for more intervention that is intensive in obesity to prevent adverse outcomes (31). The analyses in AMC, UAC, and TSF confirmed above results, although these indicators are not directly into measures of body fat or used as rough indicators of overall body fat weight and density. In addition, these indicators of body fat, just as BMI, began to decline after a certain age. Aging is associated with a substantial reduction in fat-free mass and muscle mass and the elderly are much more likely to suffer from loss of muscle mass, which plays an important etiologic role in an increased vulnerability and poor outcomes (32, 33). However, although younger generations had higher WHR and WHtR levels, both gender had a steeper increase trajectory in WHR and WHtR between ages 7 and 90, which is different from the trend of WC. WC-related values have been widely used as a representative indicator of abdominal adiposity and are all used to predict the risk of regional abdominal adiposity-related diseases (31). Recently, a Chinese research showed that WHtR is the best simple anthropometric indicator in predicting a wide range of cardiometabolic risk factors and related complications (31); and some other studies have suggested keeping one’s waist to less than half his height because WHtR has also received considerable interest (34). In our study, WHR and WHtR had a steeper increase trajectory over lifespan and Chinese younger generations had generally higher WHR and WHtR levels, which imply a very serious public health problem because of the potential risk factors of cardiovascular and related health conditions.

### Blood Pressure

Our longitudinal analyses confirmed that the mean SBP trajectory increased over lifespan in both men and women, which pattern is in line with western populations (28, 35). However, in our study, the DBP trajectory in men increased up to age 60, peaking at 83 mmHg before declining whereas for women the corresponding peak value was 82mmHg at age 80; for the Whitehall II longitudinal study 1985–2009, the mean DBP trajectory increased until age 50, and then decreased markedly in both gender, which means DBP drop appears to be starting later in life in China populations, especially among women. A widening gap between SBP and DBP develops leading to a larger and faster increase of pulse pressure with age, especially among men (36), this trend can be confirmed that the prevalence of isolated systolic hypertension (ISH) rises with aging in many studies (37). An increase of pulse pressure or DBP decline when life course approach ageing was considered as an independent determinant of developing coronary heart disease (CHD) (38).

However, even more, serious is that Chinese younger generations had higher SBP and DBP levels across adulthood, which is not consistent with the result of other western studies. In the 1985–2009 Whitehall II longitudinal study, SBP and DBP levels are generally lower for younger cohorts, and DBP decreases faster across generations compared with SBP. This opposite trend between China and these western countries may be due to the different stages of social development and capability of primary health-care systems in prevention and control of hypertension. In developed countries, pharmacological treatment, diet and lifestyle interventions, and mass education campaigns are credited for reducing the prevalence and improving the control of hypertension. In China, prevalence of hypertension rose substantially from around 20% to 34% between 2002 and 2010 (39). Accompanying rapid population ageing and continuing changes in socio-economic development, and consequential changes in lifestyle and diet over the past 30 yr; and China public health strategies mainly targeted at the promotion of pharmaceutical treatments based on hospital, proved ineffective at prevention and control of hypertension. Therefore, promoting healthier lifestyles, raising the awareness of the condition and enhancing the primary practitioner’s capability should be required to reduce the burden of blood pressure–related complications in China.

## Conclusion

We observed in a large and comprehensive longitudinal dataset that provided strong evidence of population-wide secular shifts from childhood onwards, which suggests that promoting healthier lifestyles, body weight, blood pressure and enhancing the primary practitioner’s capability should be required to reduce the burden of NCDs in China.

## Ethical Considerations

Ethical issues (Including plagiarism, informed consent, misconduct, data fabrication and/or falsification, double publication and/or submission, redundancy, etc.) have been completely observed by the authors.
